# Non-equilibrium metal oxides via reconversion chemistry in lithium-ion batteries

**DOI:** 10.1038/s41467-020-20736-6

**Published:** 2021-01-25

**Authors:** Xiao Hua, Phoebe K. Allan, Chen Gong, Philip A. Chater, Ella M. Schmidt, Harry S. Geddes, Alex W. Robertson, Peter G. Bruce, Andrew L. Goodwin

**Affiliations:** 1 Inorganic Chemistry Laboratory, University of Oxford, Oxford, OX1 3QR UK; 2 School of Chemistry, University of Birmingham, Birmingham, B15 2TT UK; 3 Department of Materials, University of Oxford, Parks Road, Oxford, OX1 3PH UK; 4 Diamond Light Source Ltd, Harwell Science and Innovation Campus, Didcot, OX11 0DE UK

**Keywords:** X-ray diffraction, Batteries, Solid-state chemistry, Structural properties, Characterization and analytical techniques

## Abstract

Binary metal oxides are attractive anode materials for lithium-ion batteries. Despite sustained effort into nanomaterials synthesis and understanding the initial discharge mechanism, the fundamental chemistry underpinning the charge and subsequent cycles—thus the reversible capacity—remains poorly understood. Here, we use in operando X-ray pair distribution function analysis combining with our recently developed analytical approach employing Metropolis Monte Carlo simulations and non-negative matrix factorisation to study the charge reaction thermodynamics of a series of Fe- and Mn-oxides. As opposed to the commonly believed conversion chemistry forming rocksalt FeO and MnO, we reveal the two oxide series topotactically transform into non-native body-centred cubic FeO and zincblende MnO via displacement-like reactions whose kinetics are governed by the mobility differences between displaced species. These renewed mechanistic insights suggest avenues for the future design of metal oxide materials as well as new material synthesis routes using electrochemically-assisted methods.

## Abstract (web summary)

The charging of Fe and Mn oxide anodes in lithium-ion batteries are believed to form rocksalt phases via reconstructive conversion reactions. Here, the authors show that *M*_*x*_O_*y*_ (*M* = Fe, Mn) transform into non-native body-centred cubic FeO and zincblende MnO via topotactic displacement-like pathways.

## Introduction

The vast majority of electrode materials operate via insertion chemistry^1^. Their performance is restricted by the homogeneity range of their crystal structures to (usually) 1*e*^−^ transfer (per formula unit) upon cycling, which limits their capacity. The search for next-generation materials with higher capacities is therefore essential to improve battery performance. As an alternative to insertion materials, conversion materials experience a complete reduction of the metal component upon discharge: $(z \cdot n){\mathrm{Li}} + {\mathrm{M}}_y{{X}}_z \leftrightarrow y{\mathrm{M}} + z{\mathrm{Li}}_n{{X}}$ (*X* = O, S, P, F, etc.). Such a reaction involves multiple-*e*^−^ transfer yielding high theoretical capacities (e.g., 1007 mAh/g for *α*-Fe_2_O_3_ c.f. 372 mAh/g for graphite)^2^ and, thus, these systems have consequently attracted considerable interest. However, the crystal structures of these materials are believed to undergo a complete de- and re-construction upon cycling—accompanied by significant volume changes that may impair the electrode’s mechanical integrity—leading to large voltage hysteresis, limited cycle life, poor rate performance and mediocre capacity retention^1,2^.

With the aim of enhancing their performance, much of the relevant research effort has focussed on material nanostructuring to promote efficient reaction with Li. In this respect, first-row transition-metal oxides (*M*_*x*_O_*y*_)—and particularly the Fe and Mn series (*M* = Fe, Mn)—have become the most studied conversion compounds due to their low cost and straightforward syntheses^3^. As a consequence, we now have a library of *M*_*x*_O_*y*_ systems with diverse nanomorphologies^4^. Although these engineered oxides have proven effective in improving rate capability and cyclability, critical issues such as low power and energy efficiency remain key obstacles to commercial application^1^. The development of viable strategies to overcome these issues therefore demands a much-improved understanding of the underlying reaction mechanisms.

**Fig. 1:**
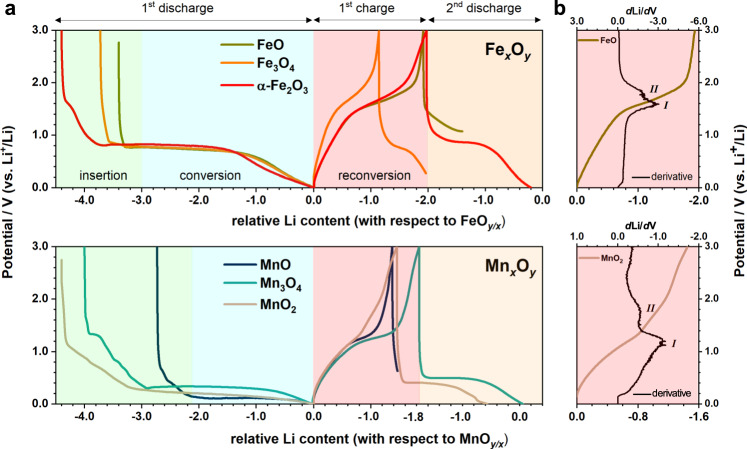
Electrochemical performance. **a** The first three cycling steps of Fe_*x*_O_*y*_ and Mn_*x*_O_*y*_ series from the in situ X-ray total scattering experiments. Distinct processes including insertion, conversion and reconversion in the respective cycling step are labelled. For easy comparison between different *M*_*x*_O_*y*_ species, the *x*-axis is against “normalised” and “relative” Li content. “Normalised” means the number of Li is weighted against the number of metal centre (*x*) in *M*_*x*_O_*y*_, i.e., the full theoretical capacity for *α*-Fe_2_O_3_ corresponds to three Li per FeO_3/2_. For the “relative” Li content, the value at the end of first discharge is set as a reference against which all other values are adjusted accordingly. **b** Derivative curves (black) of FeO and MnO_2_ plotted in parallel with their corresponding charging profile. Two distinct delithiation steps are labelled using Roman numerals.

Regardless of the particular *M*_*x*_O_*y*_ starting phase, these oxides undergo an electrochemical pulverisation during the conversion processes that leads to the formation of near-amorphous *M* and Li_2_O at the end of the first discharge^5–8^ (Fig. 1a). This *M*-Li_2_O mixture subsequently reconverts to an amorphous and/or disordered metal oxide phase (hereafter referred to as *M*_*x*’_O_*y*’_) upon charge. Despite lacking long-range order, this *M*_*x*’_O_*y*’_ was found to locally resemble a rocksalt (*rs*) type *M*O structure^5,7,9^ in both Fe and Mn systems, in turn suggesting their delithiation might follow related pathways. More importantly, the following discharge step seems to mirror the charging process, implying the actual reversible reaction might be independent on the starting *M*_*x*_O_*y*_ material. While earlier studies of these systems focussed predominantly on their initial insertion and conversion chemistry, it is critical to understand the reconversion mechanism and subsequent cycling steps, considering that these are the key processes that define the reversible capacity. Given the heterogeneous and nanoscopic nature of the (re)conversion process^5,10^, the characterisation used in previous studies primarily relied on conventional techniques such as X-ray diffraction (XRD)^5,8^, transmission electron microscopy (TEM)^9,11,12^ and X-ray absorption spectroscopy (XAS)^7,8,11^. Even with many in operando/in situ measurements undertaken, the inability of XRD to determine the short-range structure and the insensitivity of EXAFS to structure beyond the first few coordination shells^13^ means that the structural changes upon charging *M*_*x*_O_*y*_ materials remain unestablished.

In this work, we seek to address these main challenges using in operando and in situ (hereafter simplified as in situ) pair distribution function (PDF) via X-ray total scattering, a powerful tool for studying various types of conversion materials^14–16^. For a systematic and robust investigation, we have selected a series of Fe_*x*_O_*y*_ and Mn_*x*_O_*y*_ compounds and employed an analytical approach including Metropolis Monte Carlo (MMC) simulation^17^ and non-negative matrix factorisation (NMF)^18^. Our results show that these *M*_*x*_O_*y*_ systems follow an analogous cycling behaviour within a given *M* family, but the pathways differ significantly between the Fe and Mn series despite their similar electrochemical profiles during reversible cycles. Surprisingly and importantly, although a one-step conversion process from *M* to *rs*-*M*O upon charge was widely accepted in the field, we show that both Fe_*x*_O_*y*_ and Mn_*x*_O_*y*_ undergo two steps via diffusion-controlled displacement-like reactions, with O^2−^ and Mn^2+^ as displaced species, respectively, for the Fe and Mn systems forming unexpected body-centred cubic (*bcc*) FeO and zincblende (*zb*) MnO. The mechanistic transition from a conversion process to an unexpected topotactic reaction elucidates the fundamental origins of the hysteretic behaviour, offering critical insight into effective material design in the future; on the other hand, it suggests the viability of deriving new *M*O polymorphs using an electrochemically assisted approach based on battery chemistry. In addition, our study also demonstrates the value of our methodology to investigate battery material systems with complex structures.

## Results

We selected candidate Fe_*x*_O_*y*_ and Mn_*x*_O_*y*_ materials so as to include the most frequently studied compositional and crystallographic variants. The chosen Fe species were FeO^19^ (structure type: rocksalt/space group: *Fm*$\bar 3$*m*), *α*-Fe_2_O_3_^10^ (corundum/*R*$\bar 3$*c*) and Fe_3_O_4_^20^ (inverse spinel/*Fd*$\bar 3$*m*), while the Mn_*x*_O_*y*_ series consists of MnO^21^ (rocksalt/*Fm*$\bar 3$*m*), Mn_3_O_4_^22^ (distorted spinel/*I*4_1_/*amd*) and *β*-MnO_2_^23^ (rutile/*P*4_2_/*mnm*); the purity of these phases was confirmed by PDF refinement (Supplementary Fig. 1a). Note that these crystal structures are based on classic oxygen-packing frameworks, i.e., cubic-close packing (*ccp*) in *rs*-/spinel-related structure, hexagonal-close packing (*hcp*) in hexagonal corundum and tetragonal-close packing (*tcp*)^24^ in rutile. We thus expect any mechanistic similarity shared within or between the Fe and Mn families to reflect a general trend for a wider range of Fe- and Mn-oxide materials.

### Electrochemistry

Lithiation of *M*_*x*_O_*y*_ begins with an insertion step (in some materials) at a higher voltage than the conversion process (c. 0.8 and 0.3 V for Fe and Mn, respectively) and is normally accompanied by electrolyte decomposition and other side reactions^15^, which collectively give rise to the extra capacity seen in the first discharge. The following delithiation (charge) process—which corresponds to the reconversion of *M* to form the *M*_*x*’_O_*y*’_ phase—exhibits a maximum capacity of about 2 Li (per *M*) associated with 2*e*^−^ transfer (per *M*) regardless of the number of *e*^−^ involved in the initial discharge (Fig. 1a and Supplementary Fig. 1b). Discounting any side reactions after the first discharge, the removal of 2*e*^−^ per metal centre would result in a full oxidation from *M* to *M*^2+^O species. Based on a nearly identical capacity between the second discharge and the first charge, we can also infer the reconverted *M*O is the active and reversible oxide component in the following conversion–reconversion cycles. For a clearer comparison between different *M*_*x*_O_*y*_ species, the electrochemistry profiles (Fig. 1a) are plotted against the normalised “relative Li content (per *M* centre)” and will be simplified as “Li content” hereafter unless otherwise stated. It is also worth noting that although most of the *M*_*x*_O_*y*_ only exhibit one charge (pseudo-) plateau, two peaks can be resolved in their derivative curves (Fig. 1b) or cyclic voltammograms (CV)^19,23,25–28^. The two maxima are well separated in Mn_*x*_O_*y*_, but are less easily distinguished in Fe_*x*_O_*y*_. Nevertheless, their presence suggests a two-step phase transition from *M* to *M*O, deviating from the commonly-assumed single-step reaction *M* + Li_2_O ↔ *rs*-*M*O + 2Li.

### Characterisation (X-ray total scattering and TEM)

**Fig. 2:**
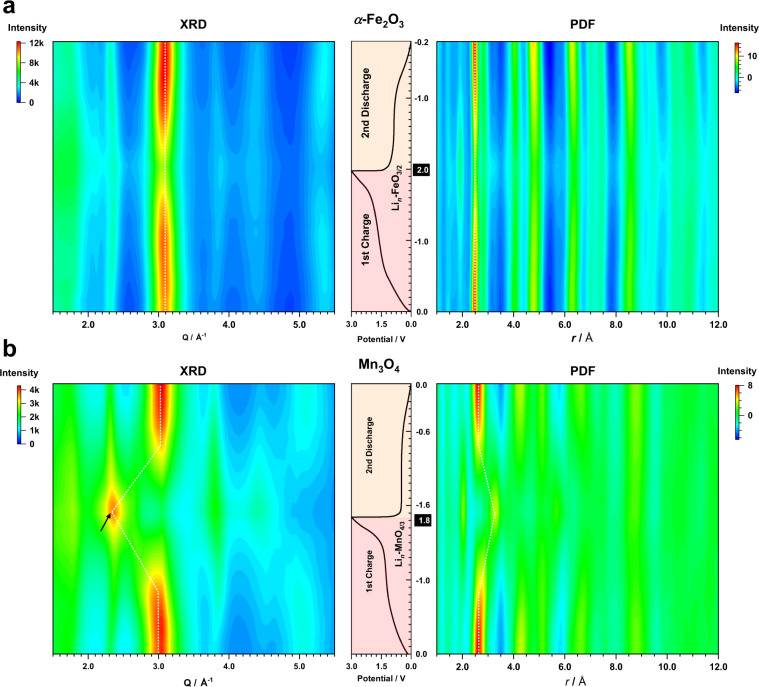
PDF and XRD patterns. Contour plot of the XRD (left) and PDF (right) patterns extracted from the in situ X-ray total scattering experiments for **a** *α*-Fe_2_O_3_ and **b** Mn_3_O_4_. Each *y*-slice of the contour plot is aligned with the corresponding “normalised relative Li content” (Fig. 1) in the first charge and second discharge cycles. The “relative Li content” at the end of the first charge is highlighted. Bragg reflection corresponding to *zb*-MnO is marked with a black arrow. The position change of the most intense peak in each contour plot is marked using a white dotted line.

Comparing the scattering patterns and PDFs (Fig. 2 and Supplementary Figs. 2–4) for different species within the same Fe_*x*_O_*y*_ or Mn_*x*_O_*y*_ series, we find that the peak evolution—i.e., peak shifts, appearance and disappearance of Bragg reflections/atom pairs—shows a single common trend. This implies comparable structure changes between different Fe_*x*_O_*y*_ or Mn_*x*_O_*y*_ systems despite their significant compositional and crystallographic differences at the beginning of first discharge. In addition, the structural change during the second discharge mirrors that during the first charge in both real and reciprocal space (Fig. 2) and the phase changes in the second charge are identical to that in the first charge (Supplementary Fig. 4b, c). This is consistent with a reversible phase behaviour. Based on the observed mechanistic resemblance within the same *M*_*x*_O_*y*_ series, we chose *α*-Fe_2_O_3_ and Mn_3_O_4_ as model compounds for subsequent studies considering that they are the most explored oxide member within each *M*_*x*_O_*y*_ family.

**Fig. 3:**
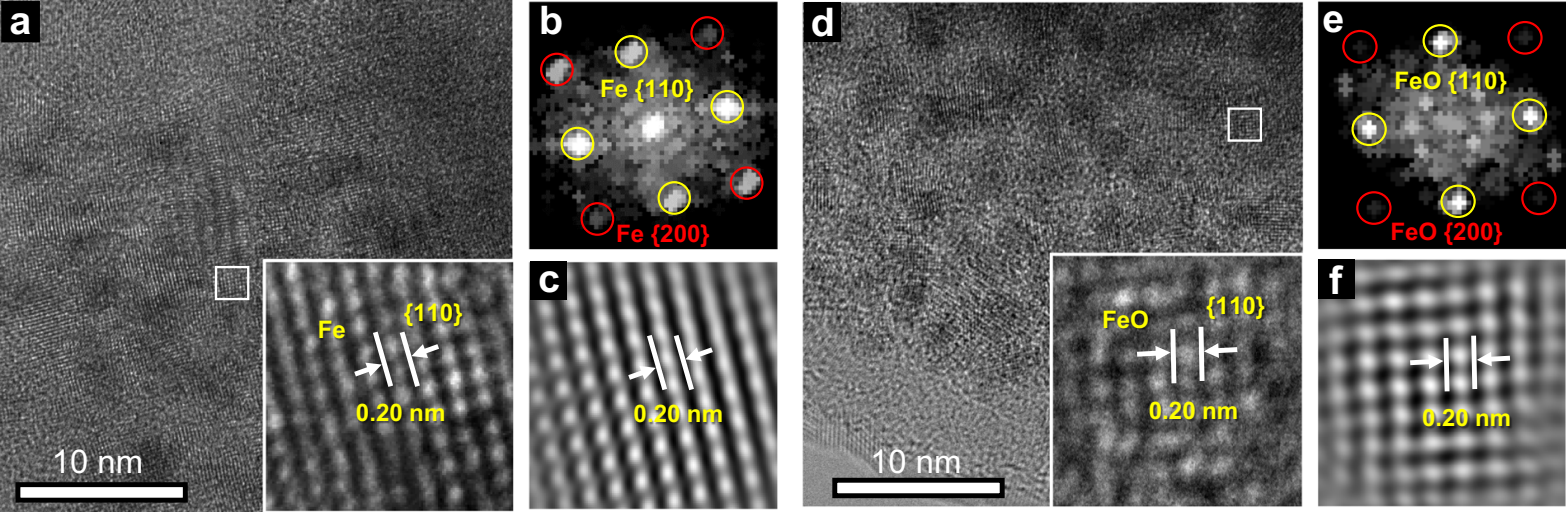
TEM data for the fully discharged and charged *α*-Fe_2_O_3_. TEM results for the *α*-Fe_2_O_3_ sample collected at **a**–**c** the beginning of charge (“Li = 0.0”) and **d**-**f** the end of charge (“Li = –2.0”). High-resolution TEM images (**a**) and (**d**) show that the *bcc*-Fe lattice is retained with {110} interlayer spacing of about 0.20 nm (Supplementary Fig. 9) in both structures (scale bar in the bottom left corner indicates 10 nm). The preservation of the *bcc*-Fe ordering is further supported by the indexing in (**b**) and (**e**) obtained from fast Fourier transform (FFT) of the highlighted region in (**a**) and (**d**). Fourier filtered images (**c**) and (**f**) based on FFT exhibit a Fe lattice comparable to the experimental pattern highlighted in (**a**) and (**d**).

To obtain better knowledge of the starting phase for the 1^st^ charge (“Li 0.0”), quantitative analysis was performed on the scattering data collected at the corresponding state of charge. As confirmed by PDF refinement (Supplementary Fig. 5) and supported by XRD simulation (Supplementary Figs. 6 and 7), the average particle size for *M* and Li_2_O phases was ∼2 and 3 nm, respectively. TEM analysis of the Fe sample (Fig. 3a–c) shows sphere-like Fe nanoparticles each a few nanometres in size. There is clear evidence of a well-ordered *bcc* lattice within nanoparticles, which can be discerned despite severe agglomeration. Upon charging, the scattering pattern evolves differently for the two systems (Fig. 2). While *α*-Fe_2_O_3_ only shows variations in peak intensities with very subtle changes in peak positions, Mn_3_O_4_ exhibits a distinct ‘waxing and waning’ effect. This contrast implies substantial mechanistic differences in the reconversion processes between the Fe_*x*_O_*y*_ and Mn_*x*_O_*y*_ series despite their similar electrochemical profiles.

### *α*-Fe_2_O_3_ (model compound for Fe_*x*_O_*y*_)

**Fig. 4:**
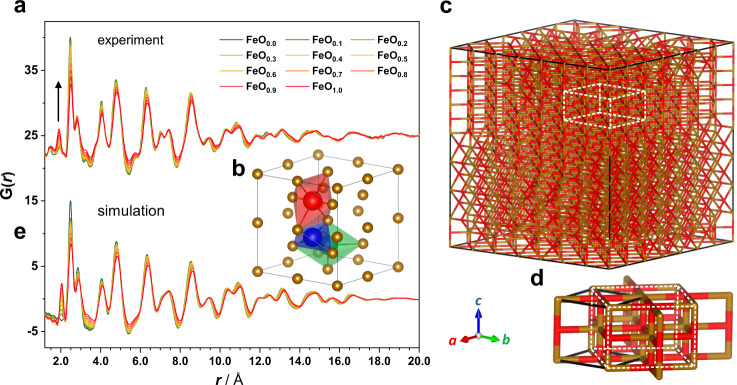
Structure modelling and PDF simulation for *bcc*-FeO_*x*_. **a** Experimental PDF patterns for *α*-Fe_2_O_3_ extracted from the first charge cycle. Colour gradient from green to red correspond to an increasing concentration of oxygen in FeO_*x*_ from *x* = 0.0 to 1.0. Black arrow indicates the growth of the 1.9 Å peak upon charge. **b** *α*-Fe structure (2 × 2 × 2) showing one *tet*-oxygen (blue) and two *oct*-oxgyens located on the face (red) and at the edge (green) of the unit cell. **c** MMC-derived FeO_1.0_ structure (7 × 7 × 7 *α*-Fe unit cell). For a clearer view, the structure is presented using a stick model (Fe: brown; O: red). **d** A repeating unit taken from an ordered domain highlighted in (**c**). The black solid frame denotes a *bcc*-FeO unit with the *I*4/*mmm* symmetry and the white dashed frame indicates a tetragonally distorted *rs*-FeO structure. **e** PDF patterns calculated using the series of FeO_*x*_ model with the same colour code shown in (**a**).

An earlier study of *α*-Fe_2_O_3_ using in situ TEM^9,11^ reported a phase transition from *α*-Fe (*Im*$\bar 3$*m*) to FeO (*Fm*$\bar 3$*m*) during the charging process. Should this reaction take place, the Fe lattice will undergo a significant rearrangement from *bcc* to *fcc* resulting in a pronounced change of the local ordering (Supplementary Fig. 8). Surprisingly, our in situ PDF data (Fig. 4a) only show subtle peak shifts with slight peak broadening and intensity reduction upon charge, implying the *α*-Fe lattice is largely retained without transformation to the *rs*-FeO. TEM measurements (Fig. 3) of the sample at the end of charge (“Li −2.0”) further confirm the retention of *bcc* Fe order in the reconverted FeO structure (hereafter denoted as *bcc*-FeO). In addition, it is also possible to discern atomic displacements (Fig. 3d and Supplementary Fig. 9) that likely account for the increased peak widths in the XRD pattern (Supplementary Fig. 6a). Remarkably, while most of the PDF atom-pair intensities progressively decrease upon charge, a peak at 1.9 Å continues to grow (Fig. 4a). Atomic distances of ~2 Å are characteristic of the first coordination sphere in Li_*n*_*M*_*x*_O_*y*_, and hence correspond to Li- or M-centred polyhedral environments. The peak cannot be ascribed to the tetrahedral Li–O in the antifluorite (*af*) Li_2_O because the concentration of this phase decreases during delithiation. Hence, we assign this peak to a Fe–O atom pair, which must correspond to O inclusion within the *bcc*-FeO phase.

The most commonly occupied interstitial site in *bcc*-Fe is the distorted octahedral site, such as is occupied by *X* = C or N in the Fe–*X* martensite alloys^29–31^. These sites are located at the centres of the faces and edges of the *bcc* unit cell (Fig. 4b). There are also 4-coordinated interstitial positions present at the 12*d* site. Both geometries are distorted compared to their *fcc*-counterparts^32^. An earlier computational study on oxygen diffusivity in *bcc*-Fe showed that the octahedrally coordinated oxygen is energetically more favourable than the tetrahedral one (hereafter denoted as *oct*- and *tet*-O, respectively)^33^. In the absence of any local structural relaxation due to the occupation of interstitial atoms, the six Fe–O pairs affiliated with the *oct*-O should contain four equatorial pairs and two short axial pairs with atomic distances of about 2.0 and 1.4 Å respectively, giving rise to an average distance of about 1.8 Å. For the *tet*-O, the four Fe–O pairs share equal distances of about 1.6 Å. Hence our experimental data, related precedent and energetic considerations all point to O inclusion within *bcc*-Fe on the octahedral interstitial site.

We developed a series of atomistic models for this *bcc*-FeO_*x*_ phase for compositions 0 ≤ *x* ≤ 1. Here the oxygen stoichiometry *x* reflects the charging capacity based on a nominal Li content “Li −2*x*”, i.e., a formula of “FeO_0.5_” corresponds to the Li content of “Li −1.0”. For selected *x* values, we introduced local structure relaxation in our models by using an MMC algorithm to incorporate simple O–O interactions. In the dilute solid solution (FeO_0.1_), the MMC-derived *oct*-O distribution appears to be disordered (Supplementary Fig. 10). However, at the high-concentration limit (FeO_1.0_), the driving force to minimise O–O repulsion energies resulted in locally ordered domains (Fig. 4c). These domains all share the same short-range structure represented by a *bcc*-FeO unit cell (Fig. 4d) that contains a face and an edge O^2‒^, isostructural to the tetragonal FeO (*I*4/*mmm*)^34^. The phase encrypts a superstructure that mirrors the tetragonally distorted *rs*-FeO where the Fe sublattice retains the *bcc* order, hinting at an underlying link between the *bcc*- and *rs*-FeO.

An important test of the physical sense of these models comes from their ability to account for the experimental PDF data. Consequently, our model PDFs were calculated using additional physical parameters, e.g., scale factor and linear correlation factor (Supplementary Fig. 11a), which were predefined via initial refinement against the Fe structure. The resulting PDFs (Fig. 4e) indeed exhibit the Fe–O peak at around 2.0 Å with growing intensity as oxygen content increases. Nearly all other variations observed experimentally as a function of composition are also well represented by our calculated PDFs. The overall agreement between simulation and experiment appears surprisingly good.

### Mn_3_O_4_ (model compound for Mn_*x*_O_*y*_)

**Fig. 5:**
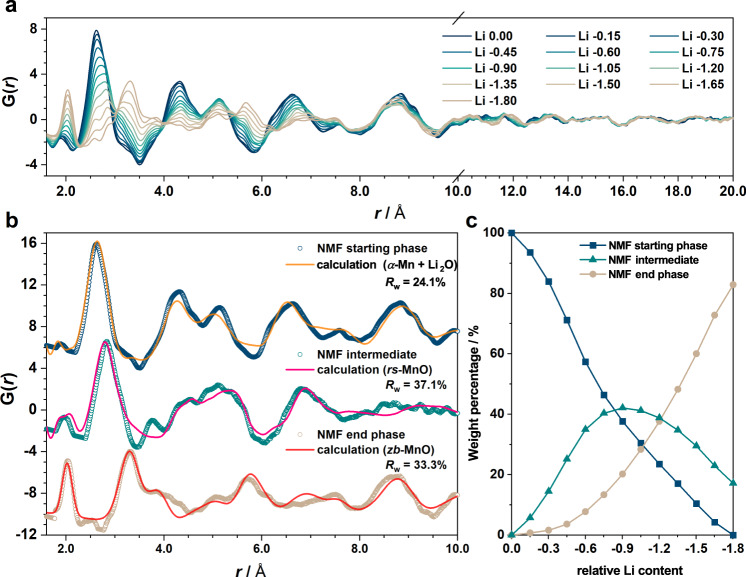
NMF analysis of the charge process of Mn_3_O_4_. **a** In situ PDF patterns for Mn_3_O_4_ during the first charge (plotted in two different *x*-scales for a clearer view). Colour gradient from dark blue (“Li 0.0”) to light brown (“Li −1.8”) corresponds to a reducing concentration of Li. **b** Three NMF-derived components in comparison with calculated PDFs using models including *α*-Mn (*I*$\bar 4$ 3 *m*), distorted *rs*-MnO (*C*2/*m*), and *zb*-MnO (*F*$\bar 43$*m*). **c** Evolution of the NMF-derived weight percentage for the components shown in (**b**).

In contrast to *α*-Fe_2_O_3_, the scattering patterns of Mn_3_O_4_ reflect the occurrence of a phase transition accompanied by significant Mn atomic rearrangement during charge. The broad width of the Bragg peaks (Supplementary Fig. 7) and the short coherence lengths (< 20 Å) of the PDF patterns (Fig. 5a) indicate that the average grain sizes of Mn-containing species remain small during delithiation. Despite a two-step electrochemical process, which suggests the involvement of an intermediate, preliminary PDF refinements were attempted using a two-phase model consisting of *α*-Mn (*I*$\bar 4$ 3 *m*) and *rs*-MnO (*Fm*$\bar 3$*m*). As anticipated, such refinements were unable to account satisfactorily for the data, demonstrating instead the necessity of including a third phase in the model. Given the analytic uncertainties concerning the intermediate, we implemented our recently developed method^18^ based on NMF^35^, a robust computational approach to study complex mixtures without a priori knowledge of the number and nature of each component. In the analysis, we employed three members each representing the starting, intermediate and end phases. The component representing the starting state was defined by the experimental pattern at the beginning of charge (“Li 0.0”), while the other two were both set as variable components to be determined by the NMF analysis. The resulting PDFs of the two unknown members (Supplementary Movie 1) both show well-resolved patterns (Fig. 5b) with their respective weightings (Fig. 5c) following systematic evolutions that resonate with the two-step mechanism. The reconstructed PDF using the NMF-derived output delivers a striking agreement with the experimental data (*R*_w_ < 1.6%), confirming the credibility of the approach (Supplementary Fig. 12).

PDF refinement was then performed to rationalise the two newly extracted components. For the reconverted phase at the end of charge, its local ordering can be well modelled using a (*zb*) MnO (*F*$\bar 43$*m*)^36^, whose (111) reflection contributes to the very intense Bragg peak at 2.3 Å^−1^ in the XRD pattern (Fig. 2). Modern computational studies^36–39^ on the polymorphic landscape of MnO highlight the relative stability of the *zb* phase^36–38^ compared to the *rs* ground-state structure; we also note the experimental discovery of a related wurtzitic polymorph^40^. Our refined lattice constant of the *zb*-MnO, 4.687 (± 0.006) Å, agrees excellently with the ab initio value 4.73 (± 0.004) Å^38^. For the intermediate component, its PDF fingerprint insinuates the Mn-containing atom pairs in an *rs*-related MnO (Supplementary Fig. 13), a phase that could also account for the scattering feature observed in the reciprocal space (Supplementary Fig.  7c). However, peak positions in real-space show discernible discrepancies implying distortion of the MnO_6_-octahedra^7^. To model this distortion, a monoclinically distorted *rs*-MnO (*C*2/*m*) was employed in the refinement, noticeably improving the fit to the NMF-derived intermediate component. Note that as a phase experiencing a transition from *α*-Mn/Li_2_O to *zb*-MnO, this intermediate is likely to remain lithiated. However, given the challenge in compositional determination, also considering the lack of structure periodicity and the likelihood of a heterogeneous cation distribution, it is difficult to reach a unique solution to fully model this intermediate. While our study concluded monoclinically distorted *rs*-MnO provides a good fit to its average short-range ordering, we cannot rule out other possible, related models.

## Discussion

Each of the phases *af*-Li_2_O, *rs*- and *zb*-MnO adopt an *fcc*-O lattice. As the Mn_*x*_O_*y*_ PDFs show little variation in the coherent length of the system—indicating comparable domain sizes of the hosting structure throughout the charge and subsequent discharge steps—we can infer the oxygen ordering likely remains essentially intact despite significant changes in the cation distribution. On the basis of this preserved oxygen framework, Mn intercalation accompanied by concurrent Li extraction (or vice versa) appears to follow a topotactic insertion-like transformation, mechanistically referred to as a displacement reaction^41^. Likewise, in the case of Fe_*x*_O_*y*_, the preservation of the *bcc*-Fe structure allows for its homogenous reaction with oxygen, implying the subsequent (dis)charge reactions of the Fe series are also diffusion-controlled, mechanistically similar to displacement, however, with O^2−^ now the displaced species. In both systems, this mechanistic transition exerts a tremendous influence on the reaction thermodynamics and kinetics of the subsequent cycles.

**Fig. 6:**
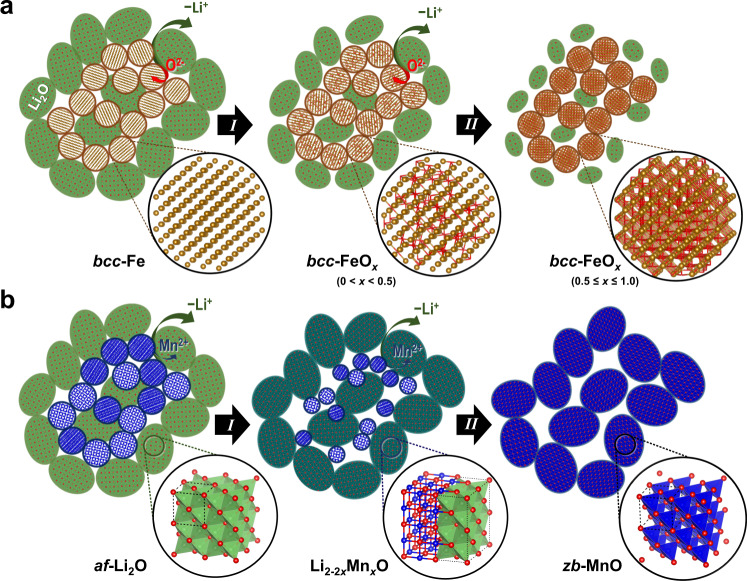
Reaction mechanism of the charge process. Illustrated mechanisms of **a** Fe_*x*_O_*y*_ and **b** Mn_*x*_O_*y*_. Host structures are highlighted and magnified in black circles. Fe, Mn, O, and Li are denoted by brown, blue, red, and green atoms/polyhedra. Yellow, blue, and green spheroids represent Fe, Mn, and Li_2_O particles, respectively. Cyan and blue ellipsoids in (**b**) represent Li_2-2*x*_Mn_*x*_O and MnO particles, respectively. Note that Li and Mn ordering in the Li_2-2*x*_Mn_*x*_O structure are shown in divided sections for a clearer view—it does not represent the actual cation distribution.

For Fe oxides, upon initial Li^+^ withdrawal from Li_2_O (process *I*), the dissociated O^2−^ migrates to the Li_2_O/Fe interface and diffuses within the Fe lattice (Fig. 6a). The inserted O^2−^ occupies the *bcc*-octahedral site, dispersing throughout the phase to reduce anion–anion repulsion. The potential presence of the Fe vacancy defects could further increase the affinity for the *oct*-O in the *bcc*-Fe^33^. As the number of inserted O^2−^ increases, the lattice expands continuously—hence a gradual shift of the Fe Bragg peaks to a lower scattering angle (Supplementary Fig.  6). In contrast, the Li_2_O reflections shift in the opposite sense due to a lattice contraction as a result of either a declining number of interstitial Li^+^ and/or reduced grain size^42^. When the oxygen content *x* reaches 0.5, the driving force to minimise the O–O interaction, hence the energy of FeO_*x*_ (Supplementary Fig. 14), promotes a disorder–order transition (process *II*) during which *oct*-O^2−^ rearranges to form an ordered sublattice. Upon further charging (*x* > 0.5), the structure becomes increasingly compact, which leads to progressively hindered O^2−^ diffusion as is consistent with the significantly increased overpotentials (Supplementary Fig. 15); ^43^ this compact structure, on the other hand, results in an increasingly strained lattice. Without evident fracture of the FeO_*x*_ nanoparticles, this strain instead manifests itself as local Fe displacements, which account for the increasingly broadened peak widths and reduced intensities observed in the PDF (Supplementary Fig. 11a). During subsequent discharge, physical parameters derived from the refinement (Supplementary Fig. 11b) exhibit a progression that echoes the evolution of the parameters obtained from the charge step. This implies a reversible two-step process occurring during the second discharge, and hence a symmetrical thermodynamic pathway between the reversible cycles.

**Fig. 7:**
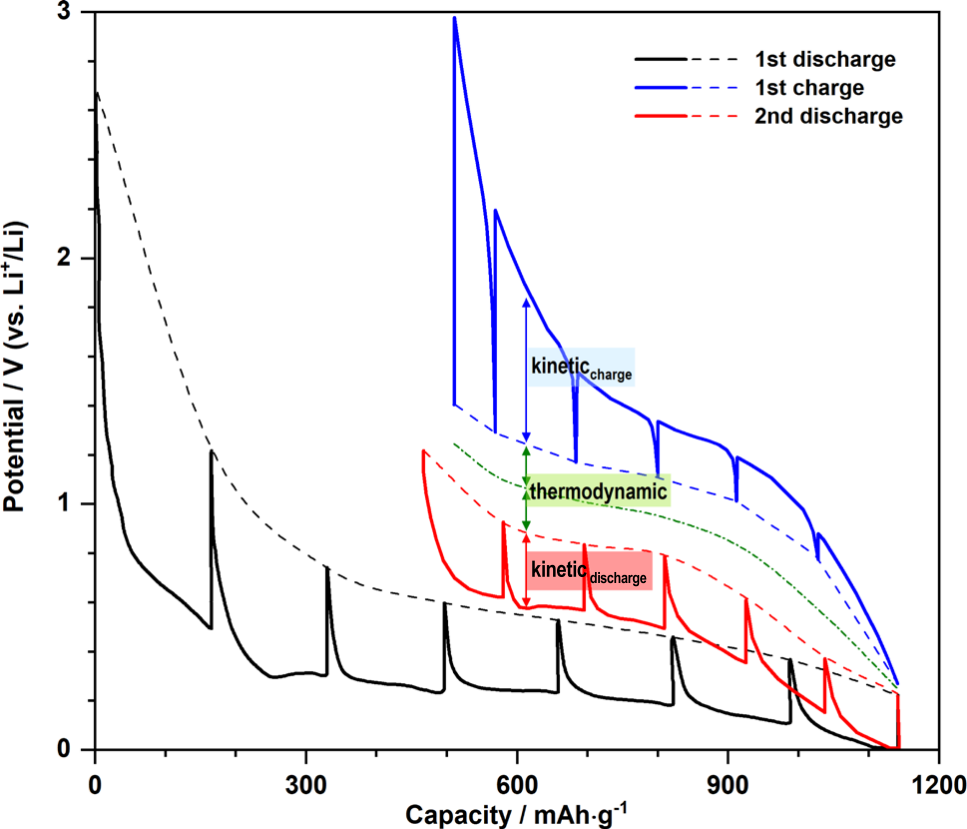
Origin of the hysteresis in *M*_*x*_O_*y*_. Galvanostatic intermittent titration (GITT) result from MnO measured by using a C/20 current rate and 64-h relaxation time (adapted from literature^21^). Solid and dash curves, respectively, denote the operating voltages and the OCVs during the first discharge (black), first charge (blue), and second discharge (red) steps. Green dash-dot curve marks the average of the OCVs between the 1^st^ charge and 2^nd^ discharge, representing the theoretical equilibrium voltage during the reversible cycles. The overpotentials upon the first charge and second discharge, which contribute to the kinetic hysteresis, are labelled by blue and red arrows, respectively. The voltage difference between the OCV (red/blue dash) and the equilibrium voltage (green dash-dot), which constitute the thermodynamic hysteresis, is labelled by green arrows.

In contrast to O^2−^ insertion into the Fe-lattice, delithiation of Mn oxides takes place via the opposite route. This difference may arise in part due to the weaker metallic bond of Mn (3d^5^4s^2^) that arises as a result of its half-filled *d*-orbitals. Upon charging, initial Li^+^ extraction partly vacates the *tet*-Li sites in *af*-Li_2_O while the oxidised Mn^2+^ dissociates from the metal nanoparticle and inserts into the octahedral vacancies in Li_2_O (Fig. 6b). This reaction (process *I*) results in an intermediate Li^tet^_2-2*x*_Mn^oct^_*x*_O phase whose cations may adopt an anisotropic distribution, rendering its average local structure similar to a distorted *rs*-MnO. Surprisingly, upon further removal of Li^+^, Mn^2+^ does not remain within the octahedral sites to form the naturally occurring *rs*-MnO; instead, it migrates to the tetrahedral positions (process *II*) where the cation ordering is as in *zb*-ZnO. During subsequent discharge cycles, based on our NMF analysis (Supplementary Figs. 16 and 17), Mn nanoparticles appear to extrude directly from the *zb*-MnO (and the remaining intermediate) without going through any traceable transition step, rendering the discharge pathway asymmetric to the charging process. For both Mn and Fe systems, based on the reaction reversibility, they should in principle exhibit identical or comparable (given the asymmetric reaction path for Mn_*x*_O_*y*_) equilibrium voltages. Thus, very similar open-circuit voltages (OCVs) between the first charge and the second discharge steps would be expected in the galvanostatic intermittent titration (GITT) plot. However, a pronounced OCV gap within the reversible cycles is evident in the experimental data (Fig. 7, marked by green arrows), reflecting a deviation from the theoretical equilibrium. Such a deviation has a thermodynamic origin and could be largely due to the voltage modification as a result of inherent “zero-current” hystersis^44^. In addition, an earlier study observed that an increase in surface tension due to the particle size reduction could increase the OCV gap^45^. Given the inevitable electrochemical grinding of *M*_*x*_O_*y*_ into ever smaller particles, their large surface energy might be another intrinsic factor contributing to this thermodynamic hysteresis.

Concerning kinetics, a displacement-like mechanism imparts a substantial dependence on the relative transport properties of different displaced species. As the discharge and charge overpotentials in both systems show an asymmetric response (Fig. 7 and Supplementary Fig. 15)—constituting a path hysteresis^46^ typical for displacement mechanism—we expect the mobilities of Mn and O in their respective host structures to be generally lower than Li diffusion in most cases. Hence, the discharge kinetics during the reversible cycles for both Mn and Fe systems is likely to be limited by Mn/O extraction from their hosts leading to a relatively small degree of variation in the discharge polarisation. By contrast, upon charge, the mobility difference between Li and Mn/O would result in more rapid Li removal than Mn/O insertion, which we observe as gradually increased overpotentials as lithium is removed. It is likely this asymmetric path hysteresis—in conjunction with other effects, such as passivation layers^11^ (hence sluggish interface mobilities)—plays a key role in modifying the voltage polarisation. The build-up of the hysteresis from both thermodynamic and kinetic origins eventually manifests itself as a voltage gap that looks unexpectedly large compared to that of conventional insertion materials. Having said that, *M*_*x*_O_*y*_’s hysteretic behaviour during the reversible cycles is notably reduced relative to that of the first cycle. It is also worth noting that the asymmetric properties in both thermodynamic (i.e., two-step charge vs one-step discharge) and kinetic pathways of the Mn system jointly give rise to its asymmetric (operating) voltage profile in which the discharge curve generally shows a prominent (pseudo-)plateau. By contrast, this effect is less pronounced in the Fe systems where both discharge and charge curves during reversible cycles appear more slope-like (Supplementary Fig. 1b).

Our results offer insights to guide the future improvement of metal oxides. Firstly, they imply that the key factors that influence the voltage polarisation are primarily mechanism-dependent—thus intrinsic to the materials. Hence, nanostructure engineering may have relatively little impact on the mitigation of the observed hysteretic behaviour^43^. That being said, *M*_*x*_O_*y*_ with unique structure designs^47^—particularly when mechanically reinforced by carbon^20,48^—might have macro- and microscopic physical features such as structural hierarchy and mesoporosity that nonetheless survive the destructive pulverisation of the first discharge. The preserved structure integrity remains important in the subsequent cycling as it could ensure both effective spatial distribution between *M* and Li_2_O as well as adequate electrolyte permeability for Li reaction. As a result, structure-engineered^47,48^ *M*_*x*_O_*y*_ is more likely to surpass non-structured^11^ materials (i.e., commercial materials) in rate performance and capacity retention (Supplementary Fig. 1b). To this end, effective morphology design remains important to harness the full potential of *M*_*x*_O_*y*_ phases. Secondly, the 2*e*^−^ transfer pathway (per *M* centre) during reversible cycling and thus the obtainable capacity (i.e., excluding the contribution from side reactions) within the regular voltage window (0–3 V vs Li^+^/Li) appear insensitive to the *M*_*x*_O_*y*_ composition. Therefore, although *M*_*x*_O_*y*_ phases with high *M* oxidation states deliver a larger capacity in the first discharge, they also produce an excess of Li_2_O, which will remain inactive during subsequent cycles. Not only is this additional capacity inaccessible thereafter, but the excess Li_2_O also exacerbates volume expansion during the first discharge—in turn impairing the mechanical integrity of the electrode^49^. Moreover, the electronically insulating effect of Li_2_O and its accumulation during the following cycles may lead to further capacity fading^11^. Hence, to effectively minimise the inherent formation of excess Li_2_O, future material development should involve strategic selection of *M*_*x*_O_*y*_ phases with lower metal oxidation states: i.e., FeO/MnO > Fe_3_O_4_/Mn_3_O_4_ > Fe_2_O_3_/Mn_2_O_3_ > MnO_2_. An alternative strategy to better control the *M*:Li_2_O ratio is to fabricate *M*/Li_2_O nano-mixtures as the pre-lithiated active material. This proof of concept has been proven viable in earlier studies where non-engineered *M*/Li_2_O nanocomposites were used as cathode additives^50^. It is worth exploring the application to anodes, with future effort directed towards carbon mixing and effective morphology engineering. Thirdly, from a broader perspective, the general interest in conversion metal oxides extends from binary to ternary phases^51^ and includes other 1st-row transition metals and beyond, e.g., Ni (*fcc*), Co (*hcp*), and Zn (*hcp*); we also noticed a change of Mn_*x*_O_*y*_ cycling behaviour in the literature due to an unintentional mixing with the Ni template^52^. In light of the mechanistic differences between the Mn and Fe systems observed here, we envisage that other binary metal oxides may exhibit quite unexpected electrochemical properties—given the distinct crystallographic and physicochemical properties of each metal. It will, therefore, be important to revisit these *M*_*x*_O_*y*_ systems (possibly with supplementary XAS techniques to enhance the knowledge of their electronic structures), such that an improved and systematic understanding of (i) their generic reversible phase behaviours upon cycling and (ii) diffusion properties of different displaced species is gained. This will uncover synergies from different *M*_*x*_O_*y*_ and hence enable the design of future materials with complex compositions that are doped with displaced species with fast mobilities, e.g., ternary systems.

As a final point we note that, while our focus has been on elucidating *M*_*x*_O_*y*_’s electrochemical transformation pathways, we do also report the first experimental observation of the non-equilibrium phases *bcc*-FeO and *zb*-MnO. In their ground states, both FeO and MnO are known to adopt the *rs* structure. Accessing other polymorphs normally requires high-temperature and/or high-pressure routes^53,54^. A recent attempt using hydrothermal methods successfully achieved nanostructured wurtzitic MnO^40^, demonstrating the viability to synthesise non-native *M*O polymorphs without extreme conditions. As a result of electrochemical pulverisation, the *M*/Li_2_O nanocomposites left after discharge possess a large surface energy. Under well-defined electrochemical conditions, this substantial surface energy can stabilise structure types that are otherwise kinetically or thermodynamically unstable, and hence allow the formation of new *M*O polymorphs. As *M*O with different structures exhibits interesting physicochemical properties, concerning the significance of *M*O in a wide range of fields, including but not limited to chemical engineering^55^, geoscience^53,54^, condensed matter physics^36,56^ and metallurgy^33,57^, the investigation of *M*O and its polymorphism has become one of the most active areas in modern solid-state chemistry. In this way, electrochemical devices may offer an alternative synthesis strategy to explore non-native metal monoxides with new functionalities—e.g., the unique formation pathway of *bcc*-FeO is based on diffusion of O^2‒^ in a close-packed Fe structure, indicating Fe nanoparticles could function as a metallic ionic conductor for O^2‒^, a rare property for Fe.

To summarise, we have used a combination of in operando/in situ PDF measurements, MMC simulations, and NMF analysis to elucidate the cycling mechanisms of Fe_*x*_O_*y*_ and Mn_*x*_O_*y*_ phases following first discharge. The mechanism adopted depends on the choice of transition metal but is insensitive to the composition of the starting *M*_*x*_O_*y*_. Although a one-step conversion reaction from *M* to *rs*-*M*O has been widely accepted as the delithiation pathway upon charge given their similar voltage profiles, our study has shown that both Fe and Mn oxides exhibit a two-step mechanism via displacement-like reactions forming non-equilibrium *bcc-*FeO and *zb-*MnO phases. Importantly, while this topotactic behaviour in Mn_*x*_O_*y*_ manifests as intercalation of Mn^2+^ into the *fcc*-O sublattice, a different pathway based on O^2‒^ insertion into the *bcc*-Fe sublattice was observed in the Fe system. For both materials—based on their distorted voltage polarisations within the reversible cycles—we show that the path hysteresis originates from the mobility differences amongst displaced species. Given the reversible cycles (within the regular voltage window) only involve 2*e*^–^ transfer (per metal centre), future material design should be directed towards systems that can demonstrate better control over excess Li_2_O produced during the first discharge. In light of the new *M*O polymorphs reported in this work, electrochemical syntheses may offer exciting opportunities to the discovery of new *M*O materials. Finally, this work demonstrates the viability of using our newly developed analytical approach combining with in situ PDF experiments to investigate battery materials with highly heterogeneous or amorphous structures.

## Methods

### Materials

All Fe_*x*_O_*y*_ and Mn_*x*_O_*y*_ materials were purchased from Sigma-Aldrich. The electrode pellets that contain 70 wt% *M*_*x*_O_*y*_ active material, 10 wt% PTFE and 20 wt% Super P carbon (TIMCAL C65) were prepared in the atmosphere. Swagelok-type in situ cells were assembled in Ar-filled glovebox (MBraun) using the as-prepared electrode pellets with a glass fibre (Whatman) as the separator, an Li metal foil as the counter electrode, and l M LiPF_6_ in ethylene carbonate (EC)/dimethyl carbonate (DMC) solution (volume ratio 1:1) as the electrolyte. The fully discharged and charged *α*-Fe_2_O_3_ materials for ex situ TEM measurement were cycled in coin cells. The coin cells were made in the same way as the in situ cells apart from the fact that *α*-Fe_2_O_3_ powder was directly employed as electrode without additional carbon or PTFE. The cells were cycled at room temperature under a rate of C/15. They were stopped at 0 and 3 V, respectively, for the discharged and charged samples. To prepare the TEM samples, these cells were transported and disassembled inside the Ar-filled glovebox. After disassembling, the materials were rinsed with DMC twice before drying in the antechamber.

### Characterisation

The in situ X-ray total scattering data were collected at beamline I15-1 at the Diamond Light Source using an amorphous silicon area detector (Perkin Elmer, XRD4343CT) with an X-ray beam of energy 76.69 keV (*λ* = 0.1617 Å) and *Q*_max_ of 25 Å^−1^. Experiments were conducted in transmission geometry within perfluoroalkoxy alkane (PFA) Swagelok cells. Empty Swagelok cells were measured for the background of every in situ cell. The in situ electrochemistry was conducted using a rate of C/15 for every cell. Data reduction and normalisation were performed using DAWN^58^ and PDFgetX2^59^, respectively. An additional LaB_6_ pattern was collected as a reference to obtain instrumental damping factor for PDF refinement. The PDF refinements were performed using the PDFgui^60^ software. For TEM measurements immediately following preparation of the ex situ samples, we used a JEOL 3000 F Field emission gun TEM with an air-tight TEM holder.

### MMC simulation

MMC simulations^17^ were performed on a supercell constructed using 7 × 7 × 7 *α*-Fe unit cell with the periodic boundary conditions applied. The dimensions of the box were determined based on the 2 nm size of the *α*-Fe particles suggested by PDF. The number of oxygen atoms was determined by the Fe:O ratio in FeO_*x*_ and were first randomly distributed into the *oct*-sites of the *bcc*-Fe. The MMC energy was calculated on the basis of repulsion between oxygen considering the O–O interaction within the three nearest coordination shells of each oxygen. At each MMC step, a randomly selected oxygen atom was swapped with another randomly selected empty *oct*-site. The energy change (Δ*E*) due to the move was calculated. The move was automatically accepted if Δ*E* ≤ 0; for a positive energy change, the acceptance was subject to the Metropolis algorithm^17^ given by ${{P}} = {{e}}^{ - \Delta {\it{E}}/{\it{kT}}}$, where *k* is the Boltzmann constant and *T* is the temperature. Moves were continuously proposed, and accepted or rejected until convergence was achieved.

### Non-negative matrix factorisation

The NMF approach followed closely the Metropolis Matrix Factorisation (MMF) method reported earlier^18^, which uses the MMC algorithm to carry out NMF^35^. The NMF analysis was performed on renormalised PDFs to satisfy the non-negative criterion of NMF. The renormalised $g^{{\mathrm{exp}}}\left( r \right)$ were derived from the experimental $G^{{\mathrm{exp}}}\left( r \right)$ using the equation $G\left( r \right) = 4\pi r\rho _0({\mathrm{g}}\left( r \right) - 1)$^61^, in which *ρ*_0_ refers to the number density of the structure model. Three fundamental components ${g \ast}_{{\hskip -6pt} i} \left( r \right)$ (*i* = 3) were employed in the analysis. The goal of the analysis was to identify these ${g \ast}_{{\hskip -6pt} i} \left( r \right)$ and associated weights *w*_*ij*_ (*j* corresponds to the number of experimental $g_j^{{\mathrm{exp}}}\left( r \right)$) to minimise $|g_j^{{\mathrm{calc}}}\left( r \right) - g_j^{{\mathrm{exp}}}\left( r \right)|^2$, where $g_j^{{\mathrm{calc}}}\left( r \right) = {\sum}_{i{\mathrm{ = 1}}}^3 {w_{{\mathrm{ij}}}{g \ast}_{{\hskip -6pt} i} \left( r \right)}$. Additional constraints were applied to ensure non-negative ${g \ast}_{{\hskip -6pt} i} \left( r \right)$ for all *i* and *r*, and that ${\sum }_{i{\mathrm{ = 1}}}^3 w_{{\mathrm{ij}}}{\mathrm{ = 1}}$ for all *j*. The initial ${g \ast}_{{\hskip -6pt} 1} \left( r \right)$ representing the known component was fixed as the experimental function measured at the end of first discharge, whereas the two unknown components ${g \ast}_{{\hskip -6pt} 2} \left( r \right)$ and ${g \ast}_{{\hskip -6pt} 3} \left( r \right)$ and all *w*_*ij*_ were assigned randomly subject to the various constraints listed above. Each iteration involved a random variation of these parameters, followed by the calculation of the change in $|g_j^{{\mathrm{calc}}}\left( r \right) - g_j^{{\mathrm{exp}}}\left( r \right)|^2$. The acceptance or rejection of the variation follows MMC algorithm. The variation was repeated under increasingly stringent acceptance criteria using simulated annealing until convergence was achieved.

## Supplementary information

Supplementary Information

Description of Additional Supplementary Files

Supplementary Movie 1

## Data Availability

The authors declare that all data supporting the findings of this study are included within the paper and its Supplementary Information files. Source data are available from the corresponding author upon reasonable request.
