# Supplementary material for: Non-equilibrium metal oxides via reconversion chemistry in lithium-ion batteries
Source: Nat Commun. 2021 Jan 25;12:561. doi: 10.1038/s41467-020-20736-6 (PMC7835223; doi:10.1038/s41467-020-20736-6)
Supplement: Supplementary file 1 — Supplementary Information [file 41467_2020_20736_MOESM1_ESM.pdf]

SUPPLEMENTARY INFORMATION

Non-equilibrium Metal Oxides via Reconversion Chemistry in Lithium-ion Batteries

Xiao Hua[1, *], *Phoebe K. Allan*[2], *Chen Gong*[3], *Philip A. Chater*[4], *Ella M. Schmidt*[1], *Harry S. Geddes*[1], *Alex W. Robertson*[3], *Peter G. Bruce*[3], *Andrew L. Goodwin*[1]

[1] Inorganic Chemistry Laboratory, University of Oxford, Oxford OX1 3QR, UK

[2]School of Chemistry, University of Birmingham, Birmingham B15 2TT, UK

[3]Department of Materials, University of Oxford, Parks Road, Oxford OX1 3PH, UK

[4]Diamond Light Source Ltd, Harwell Science and Innovation Campus, Didcot OX11 0DE, UK

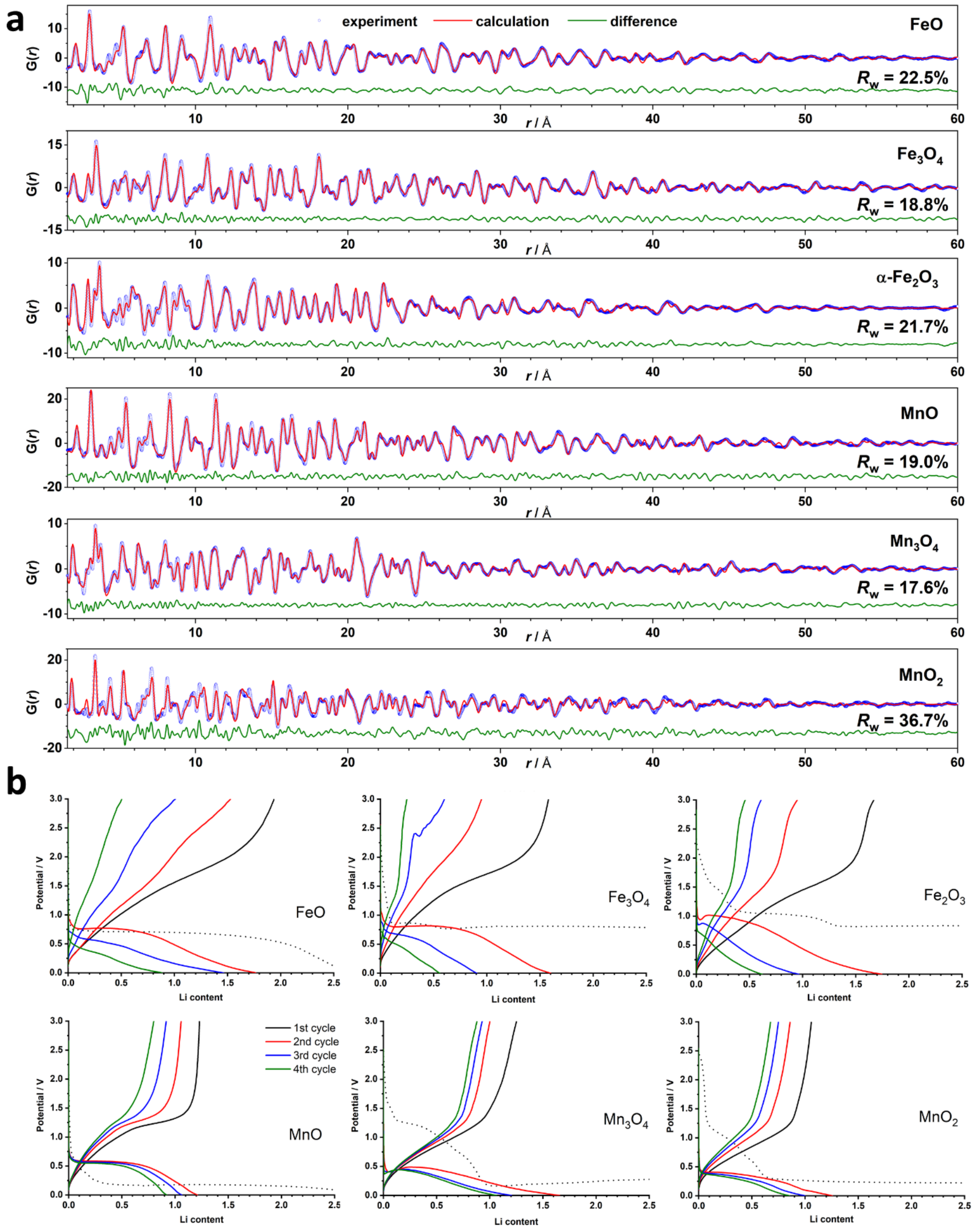

Supplementary Figure 1. a) PDF refinement of the starting M_xO_y phases revealing particle sizes of > 7 nm for all the samples. **b)** The first four cycles of M_xO_y. All first discharge profiles are plotted in dotted lines with the regions corresponding to the extended capacities (Li > 2.5) omitted for a clearer view.

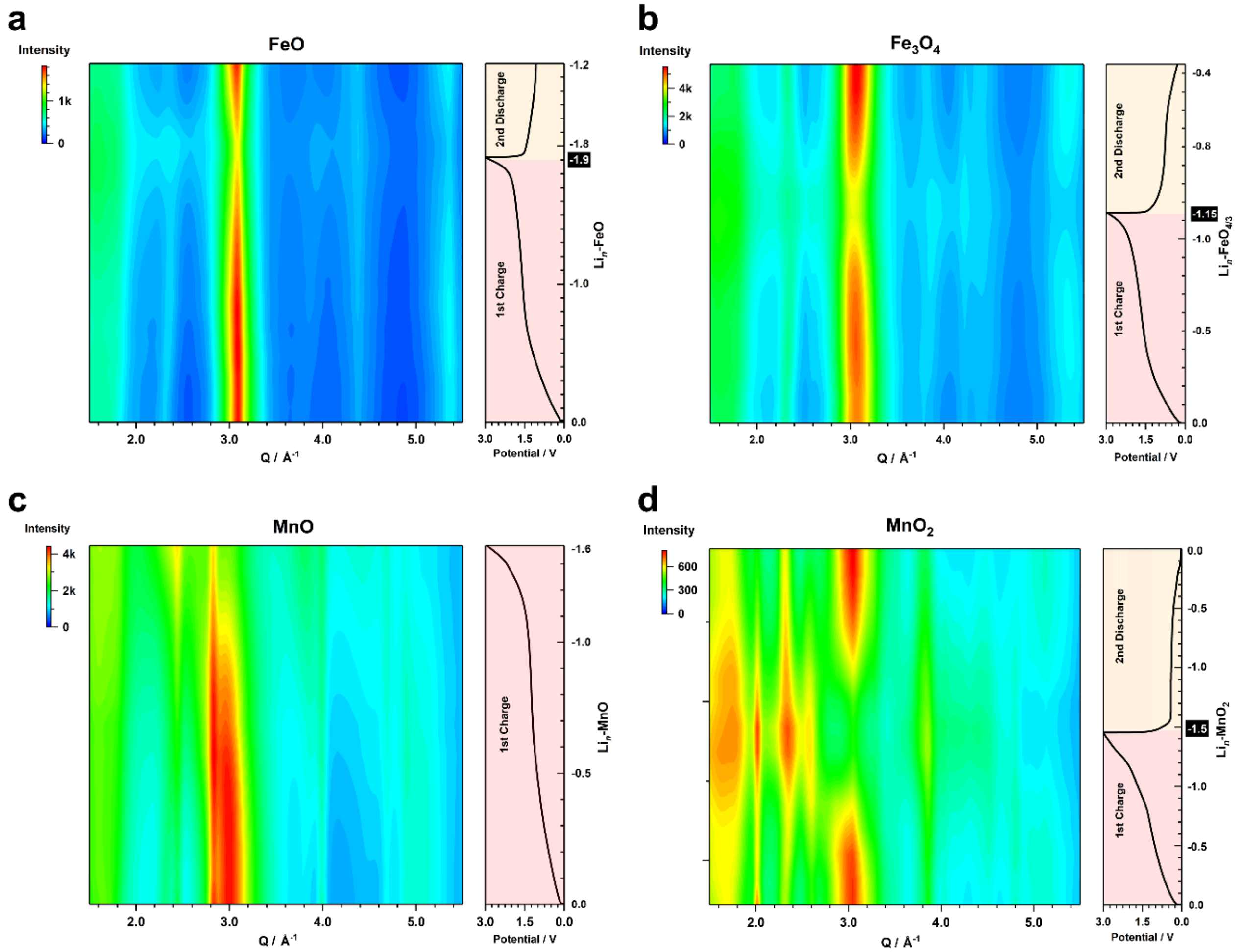

Supplementary Figure 2. Contour plot of the XRD patterns extracted from the *in situ* X-ray total scattering experiments for **a)** FeO, **b)** Fe_3O_4, **c)** MnO, and **d)** β-MnO_2. Each y-slice of the contour plot is aligned with the corresponding "normalised relative Li content" (Fig. 1) in the first charge and second discharge cycles. The "relative Li content" at the end of first charge is highlighted.

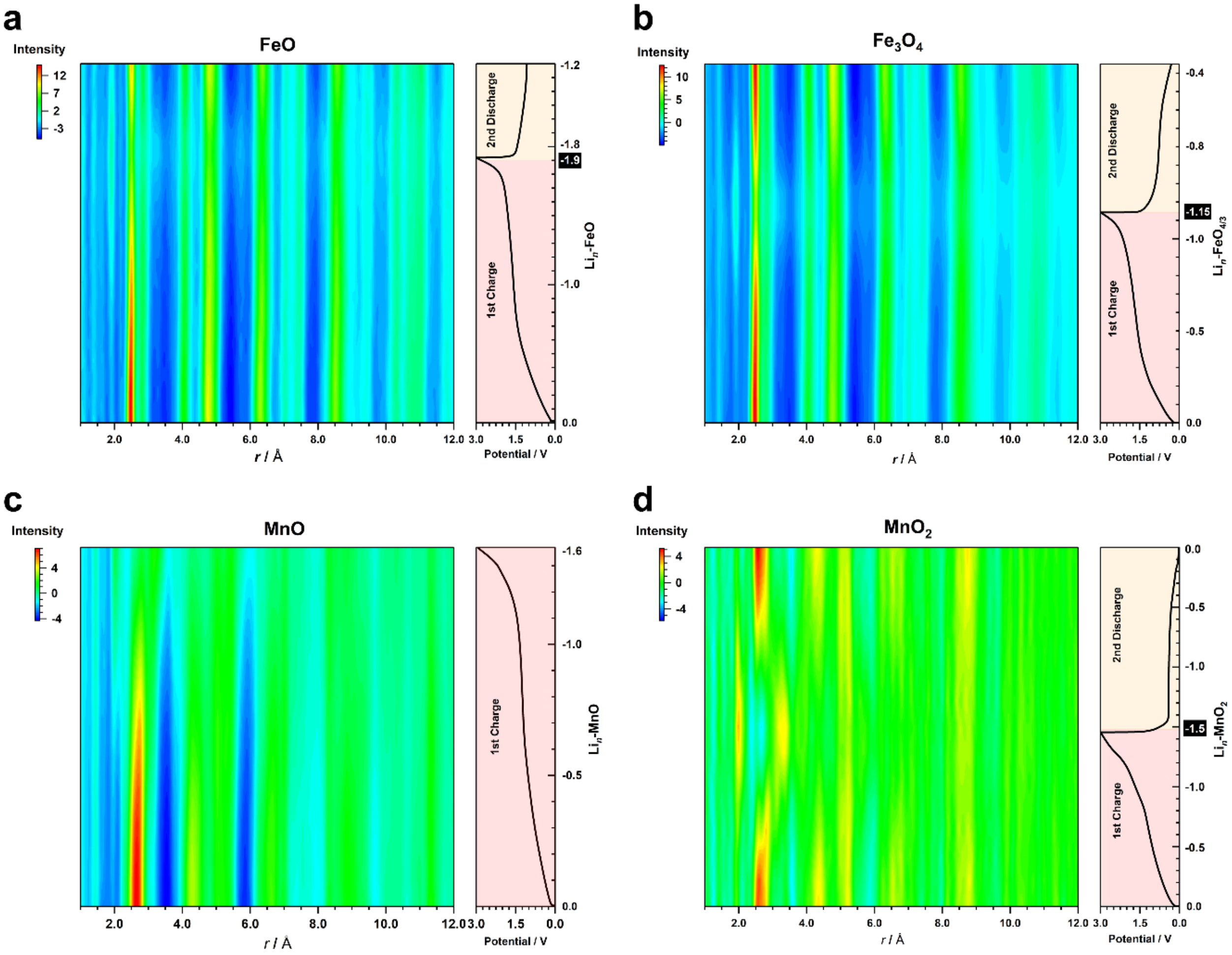

Supplementary Figure 3. Contour plot of the PDF patterns extracted from the *in situ* X-ray total scattering experiments for **a)** FeO, **b)** Fe_3O_4, **c)** MnO, and **d)** β-MnO_2. Each *y*-slice of the contour plot is aligned with the corresponding "normalised relative Li content" (Fig. 1) in the first charge and second discharge cycles. The "relative Li content" at the end of first charge is highlighted.

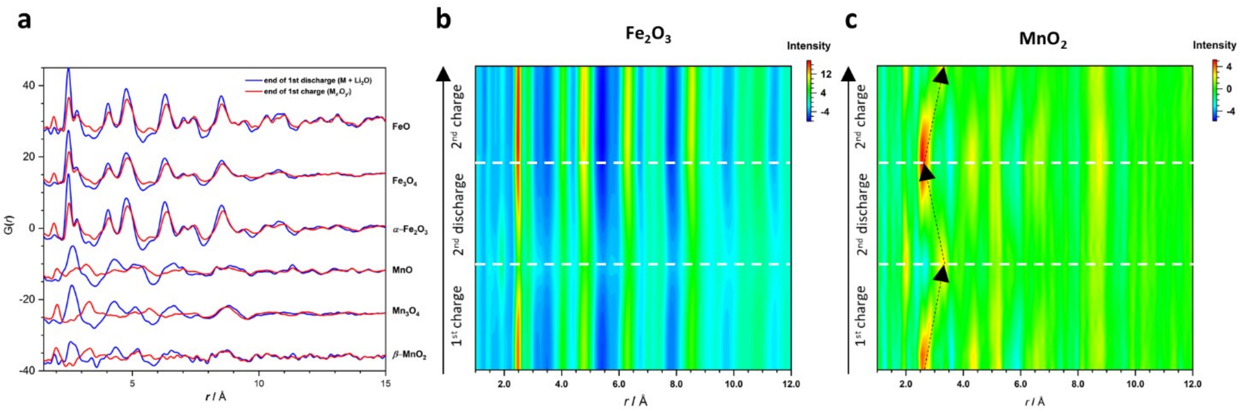

Supplementary Figure 4. a) Selected PDF patterns extracted from the *in situ* X-ray total scattering experiments at the end of first discharge (blue) and the end of first charge (red) for series of M_xO_y. Highly similar patterns are exhibited in each M_xO_y series which implies their analogous phase transformation pathways. Contour plots of the PDF data for **b)** α-Fe_2O_3 and **c)** MnO_2 during the 1st charge, 2nd discharge and 2nd charge cycles (separated by white dashed lines) show that the phase behaviours of the 2nd charge in both Fe and Mn systems are identical to that of the 1st charge, confirming the reversibility of the mechanism elucidated in this study. Notable peak changes in MnO_2 are indicated by dashed arrows.

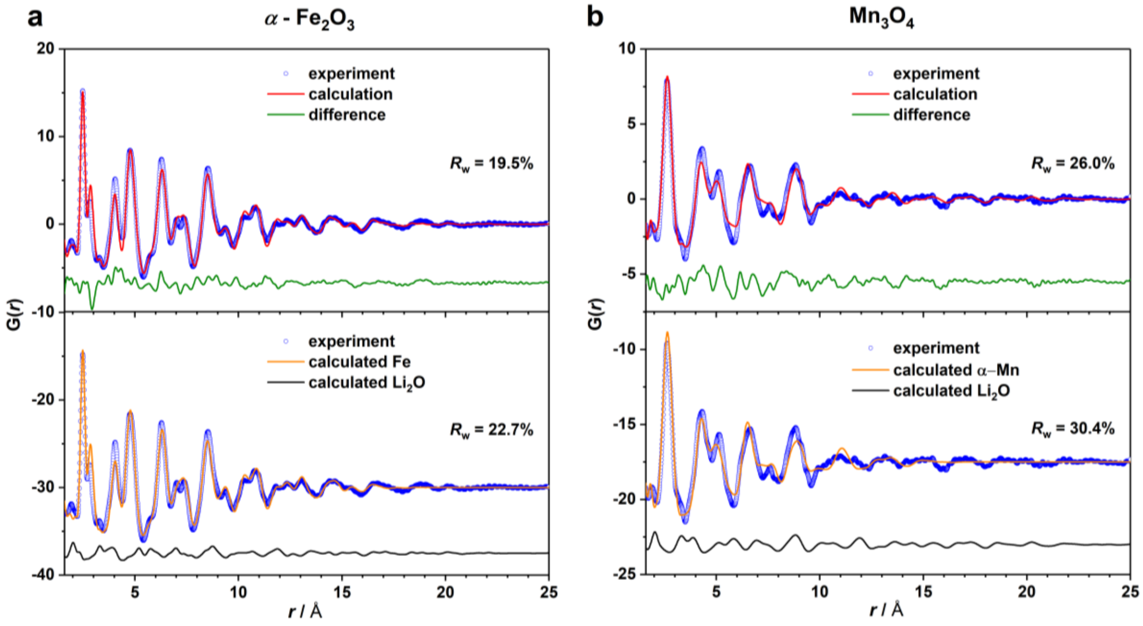

Supplementary Figure 5. a) PDF refinement of α-Fe_2O_3 collected at the beginning of charge ("Li 0.0") using α-Fe + Li_2O model whose individual contribution is shown separately in the bottom. The refinement generated particle sizes of 19.86 (± 0.03) and 28.28 (± 0.40) Å for Fe and Li_2O, respectively. The agreement factor (R_w) with or without including Li_2O in the model is 19.5% and 22.7%, respectively. **b**) PDF refinement of Mn_3O_4 collected at the beginning of charge ("Li 0.0") using α-Mn + Li_2O model whose individual contribution is shown separately in the bottom. The refinement generated particle sizes of 17.67 (± 0.07) and 33.07 (± 0.78) Å for α-Mn and Li_2O, respectively. The R_w with or without including Li_2O is 26.0% and 30.4%, respectively.

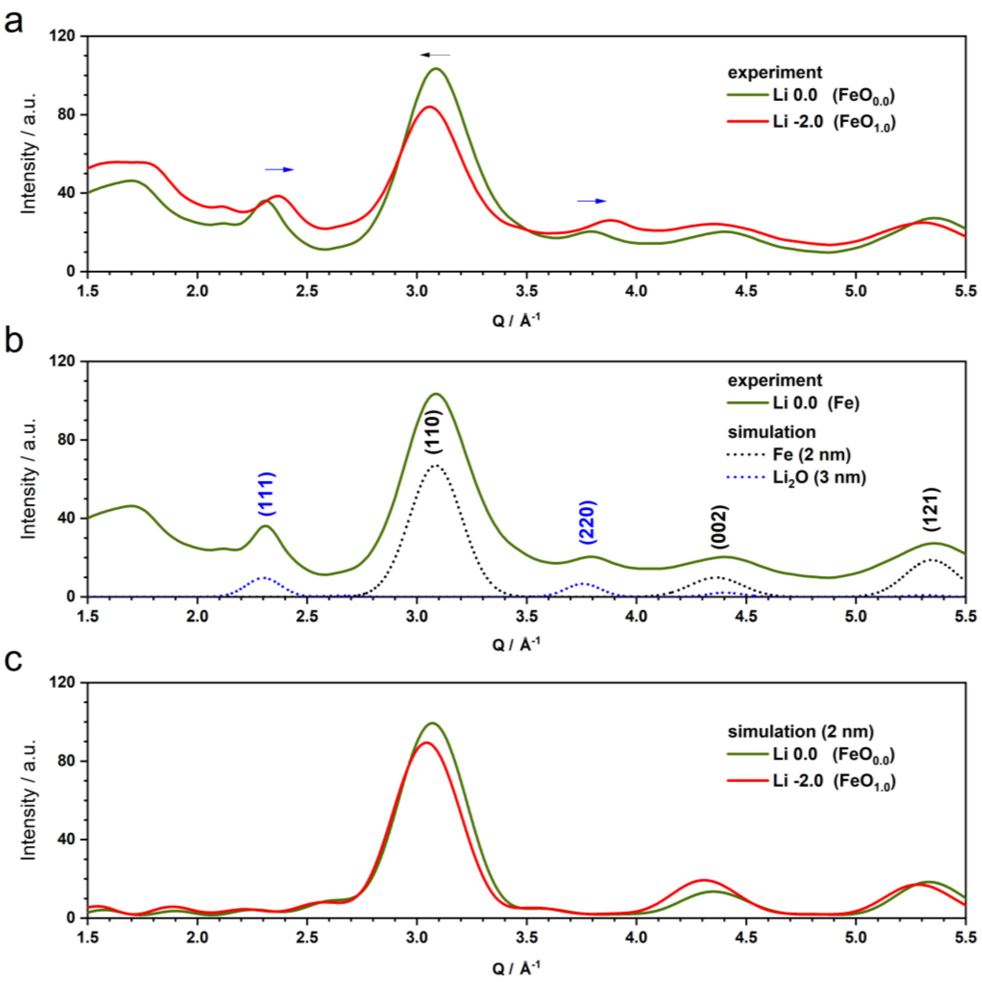

Supplementary Figure 6. a) Experimental XRD of α-Fe_2O_3 collected at the beginning ("Li 0.0") and the end ("Li -2.0") of the first charge process. Black and blue arrows indicate the Bragg shifts of the Fe and Li_2O phases, respectively. **b)** Comparison of the XRD at the beginning of charge ("Li 0.0") between experimental data and calculated pattern using the model composed of Fe (2 nm) and Li_2O (3 nm) phases. The sizes are obtained from PDF refinement of the same state of charge (Fig. S5a). **c)** Calculated XRD pattern using the MMC-derived FeO_x structure.

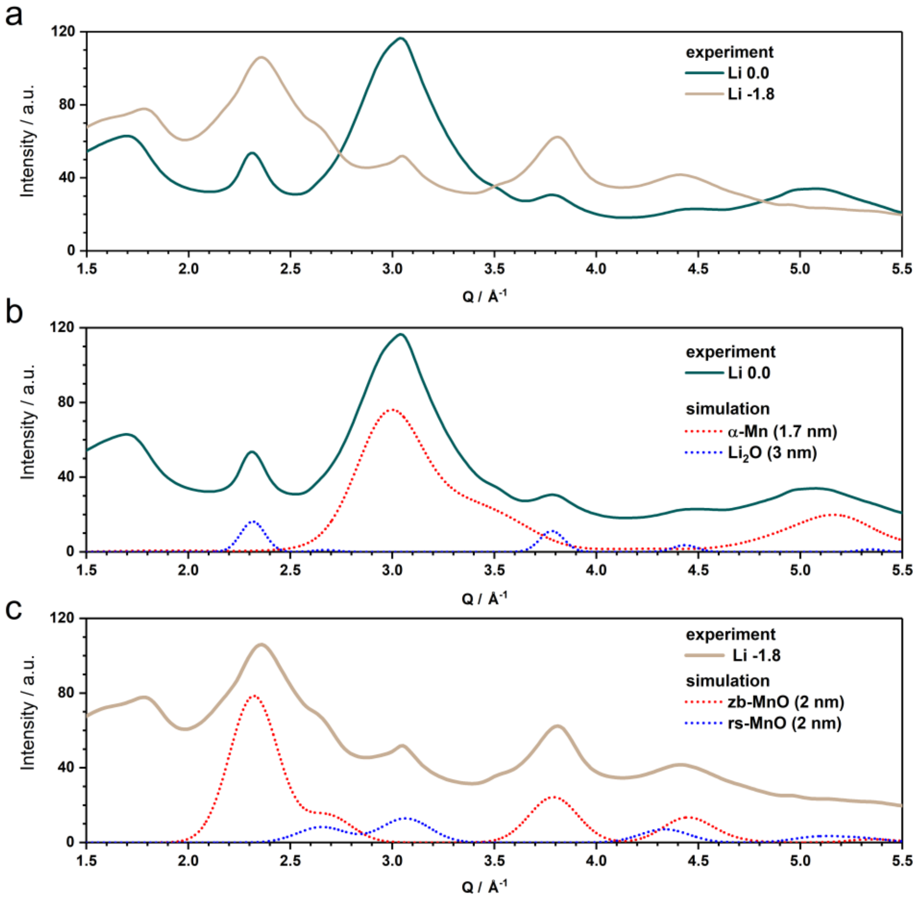

Supplementary Figure 7. a) Experimental XRD of Mn_3O_4 collected at the beginning ("Li 0.0") and the end ("Li -1.8") of the first charge process. **b)** Comparison of the XRD at the beginning of charge ("Li 0.0") between experimental data and calculated pattern using the model composed of Mn (1.7 nm) and Li_2O (3 nm) phases. The sizes are obtained from PDF refinement of the same state of charge (Fig. S5b). **c)** Comparison of the XRD at the end of charge ("Li -1.8") between experimental data and calculated pattern using the NMF-derived model containing *zb*- and distorted *rs*-MnO phases.

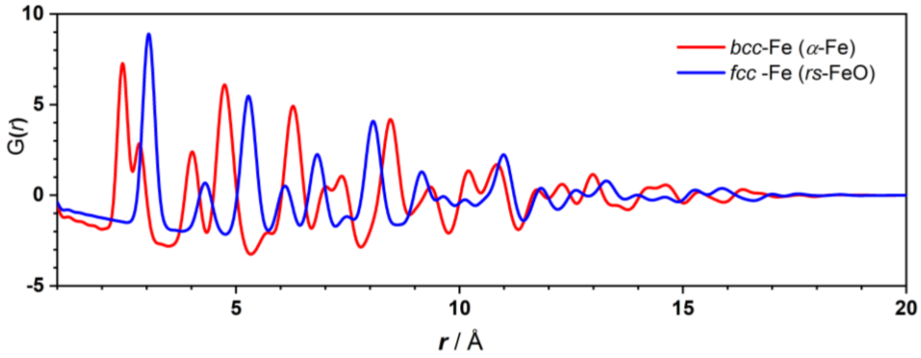

Supplementary Figure 8. Comparison of computed PDF contribution from the *bcc*-Fe lattice in *α*-Fe (red) and *fcc*-Fe lattice in *rs*-FeO (blue).

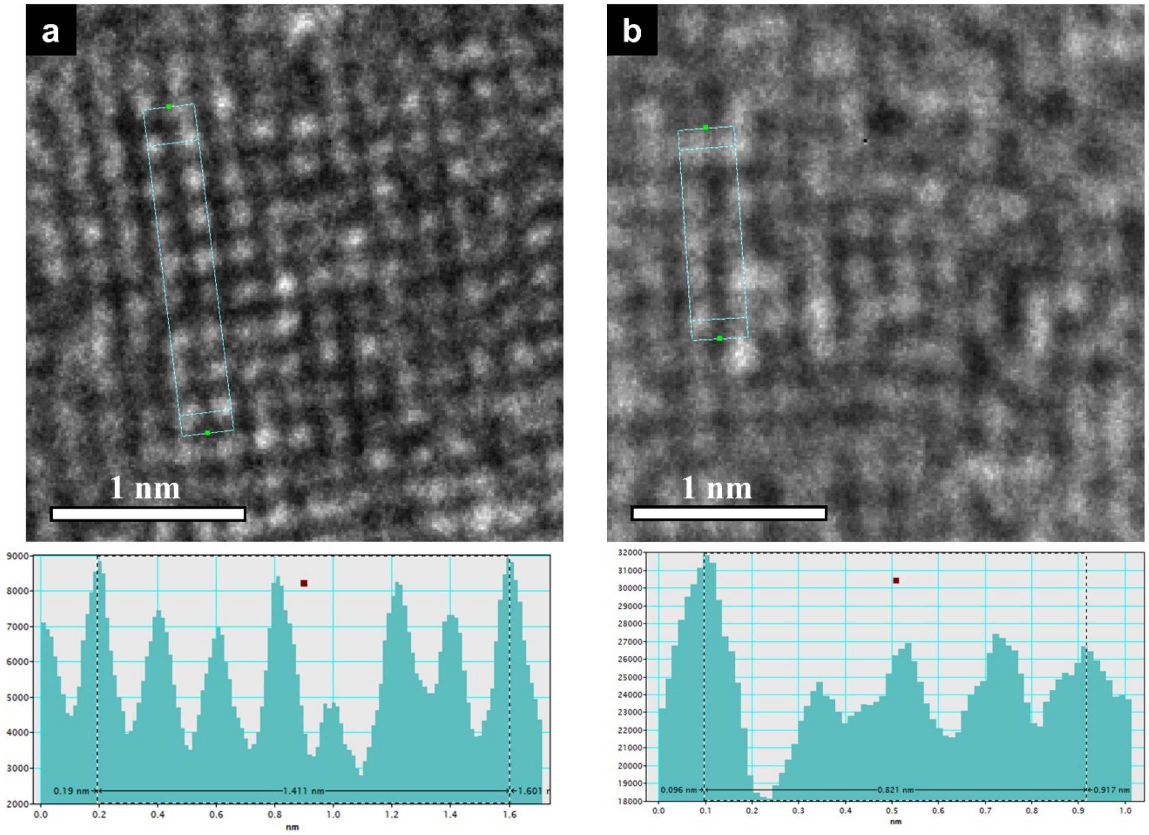

Supplementary Figure 9. HRTEM images of *α*-Fe_2O_3 sample collected at **a)** the beginning ("Li = 0.0") and **b)** the end ("Li = -2.0") of the first charge process. Highlighted region (blue dashed box) was used to extract average lattice spacing for each sample. More defected Fe lattice is evident in b) comparing to that in a).

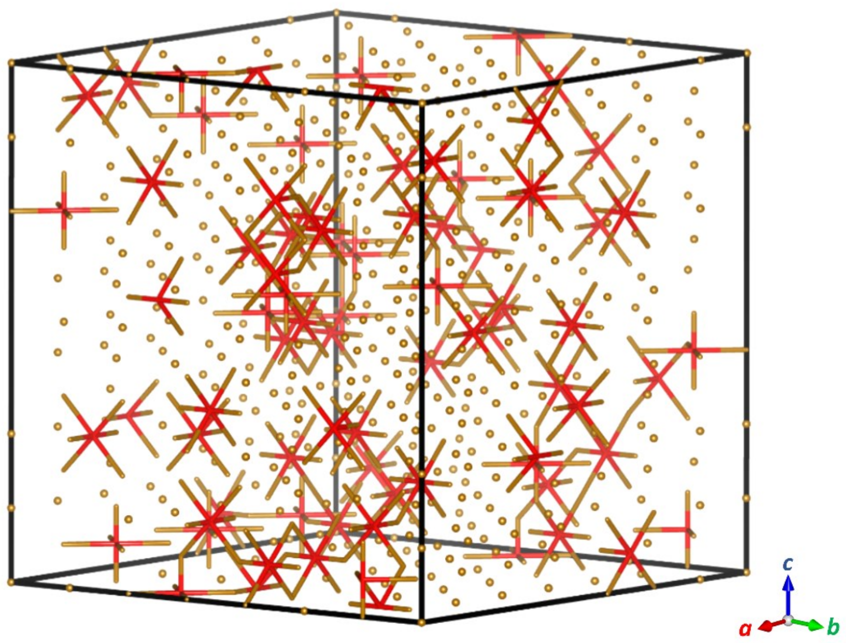

Supplementary Figure 10. MMC-derived FeO_x structure with dilute oxygen concentration (x = 0.1). For a clearer view, bonded Fe and O are represented using stick model with atoms omitted.

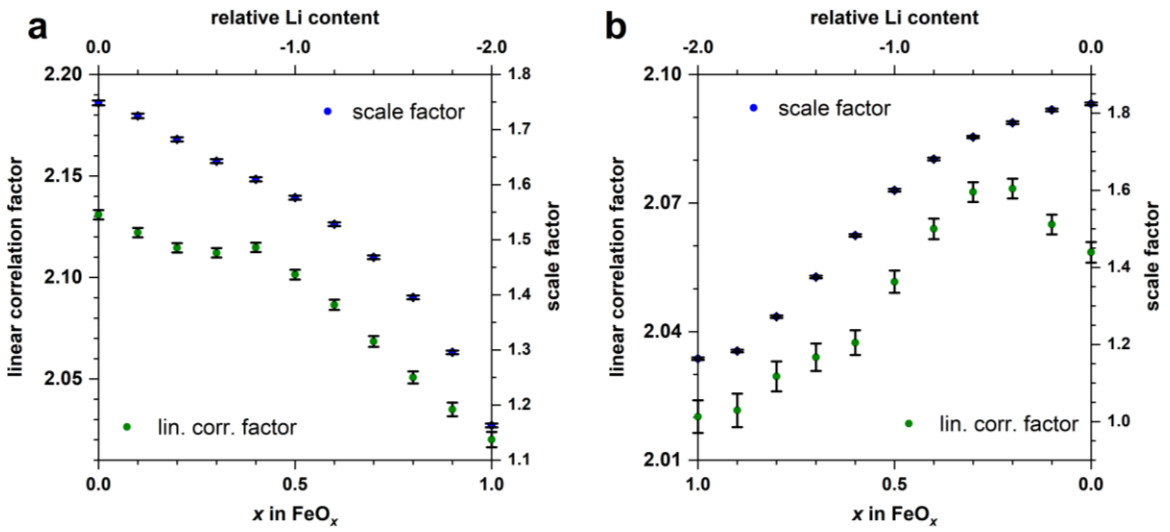

Supplementary Figure 11. Refined scale factor (blue circle) and linear correlation factor (green circle) obtained from refinement of α-Fe_2O_3 PDF data collected during **a)** the first charge and **b)** the second discharge processes using α-Fe structure. Error bars (black) that reflect the standard deviations derived from the refinements are also shown.

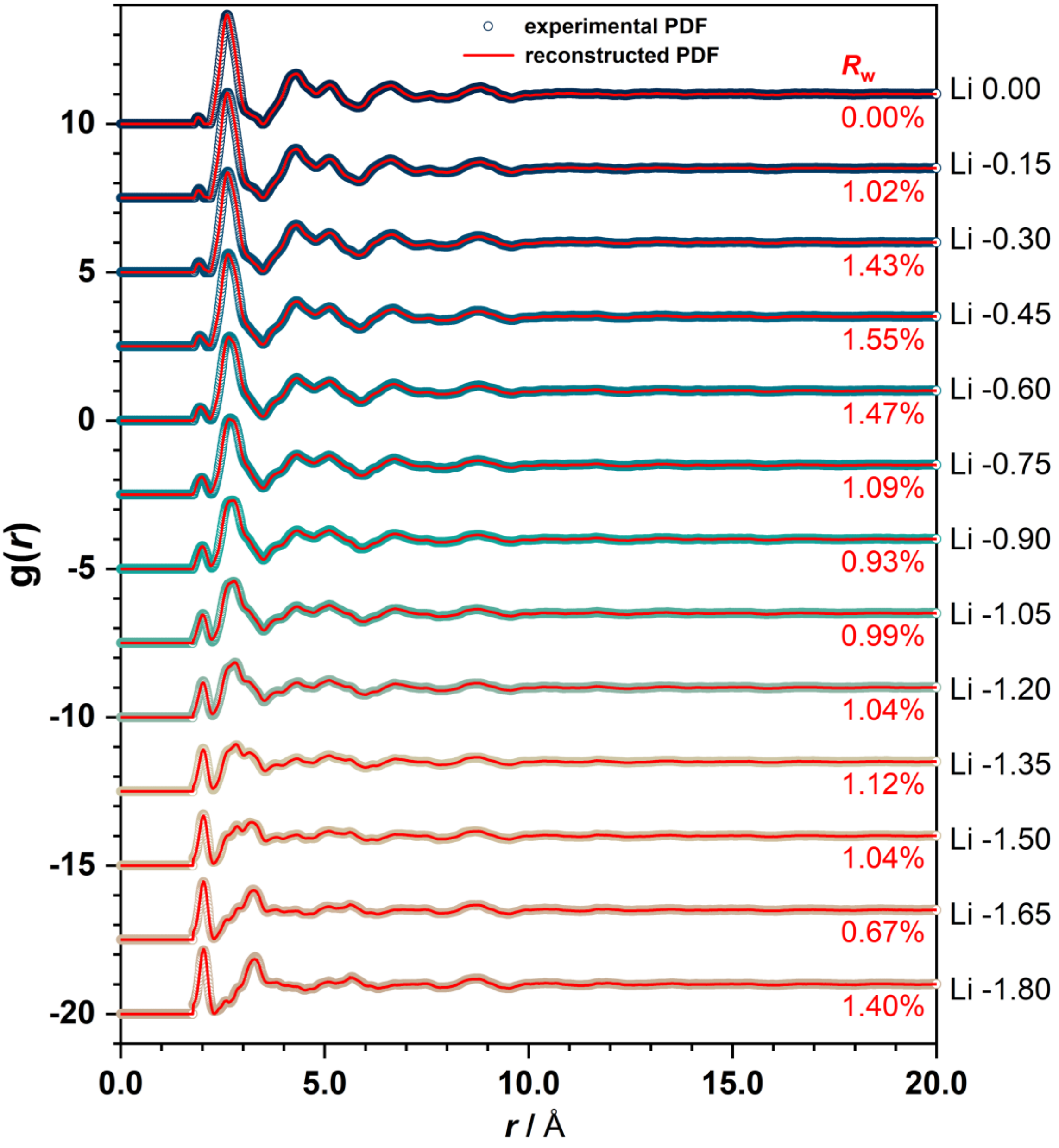

Supplementary Figure 12. Comparison between the experimental PDF from the first charge (with the same colour gradient shown in Fig. 5) and the PDF reconstructed (red) using NMF-derived components and their respective weightings. R_w agreement factors are labelled in red.

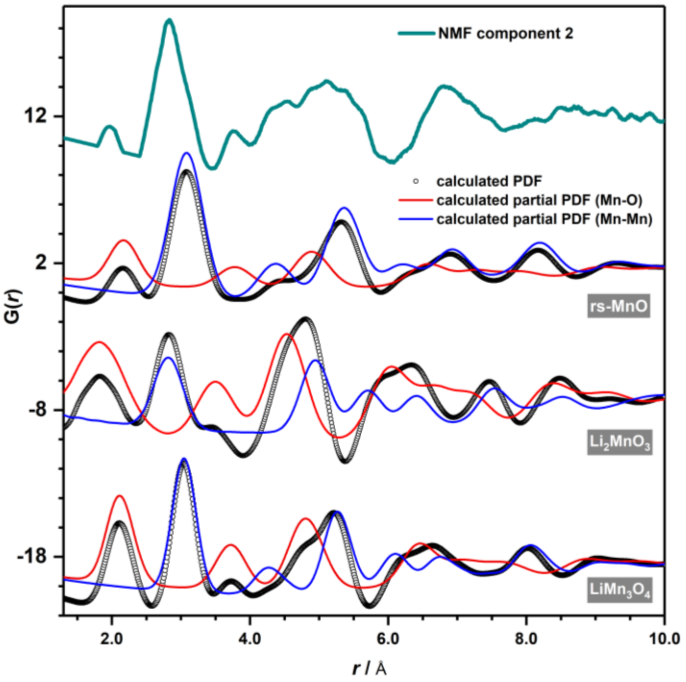

Supplementary Figure 13. NMF-derived component 2 (intermediate component) in comparison with calculated PDFs using selected *rs*-related Mn-containing oxides including *rs*-MnO ($Fm\bar{3}m$), Li_2MnO_3 ($C2/c$), $LiMn_3O_4$ ($C2/m$). Contributions from Mn-O (red) and Mn-Mn (blue) atom pairs are also shown.

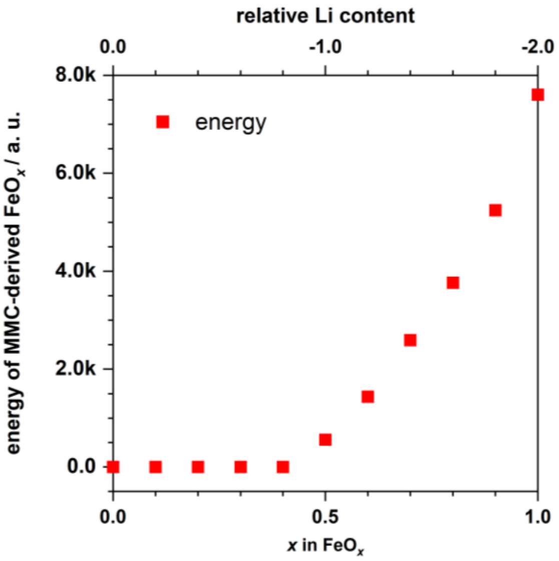

Supplementary Figure 14. Evolution of the calculated energy for the MMC-derived FeO_x structures.

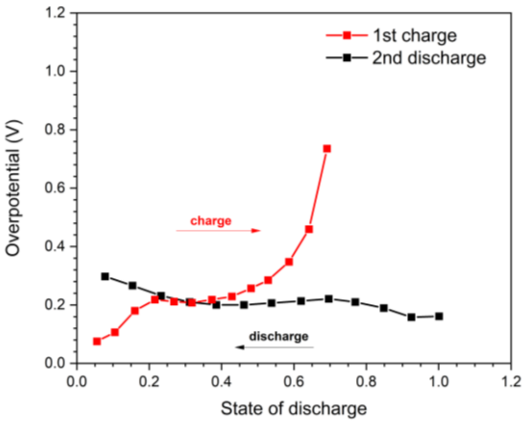

Supplementary Figure 15. Overpotential derived from the GITT result from the second cycle of α-Fe_2O_3 (adapted from literature[1]) including the 1st charge (red) and 2nd discharge (black). The red curve corresponding to the charge reaction shows a growing overpotential with increasing "state of discharge".

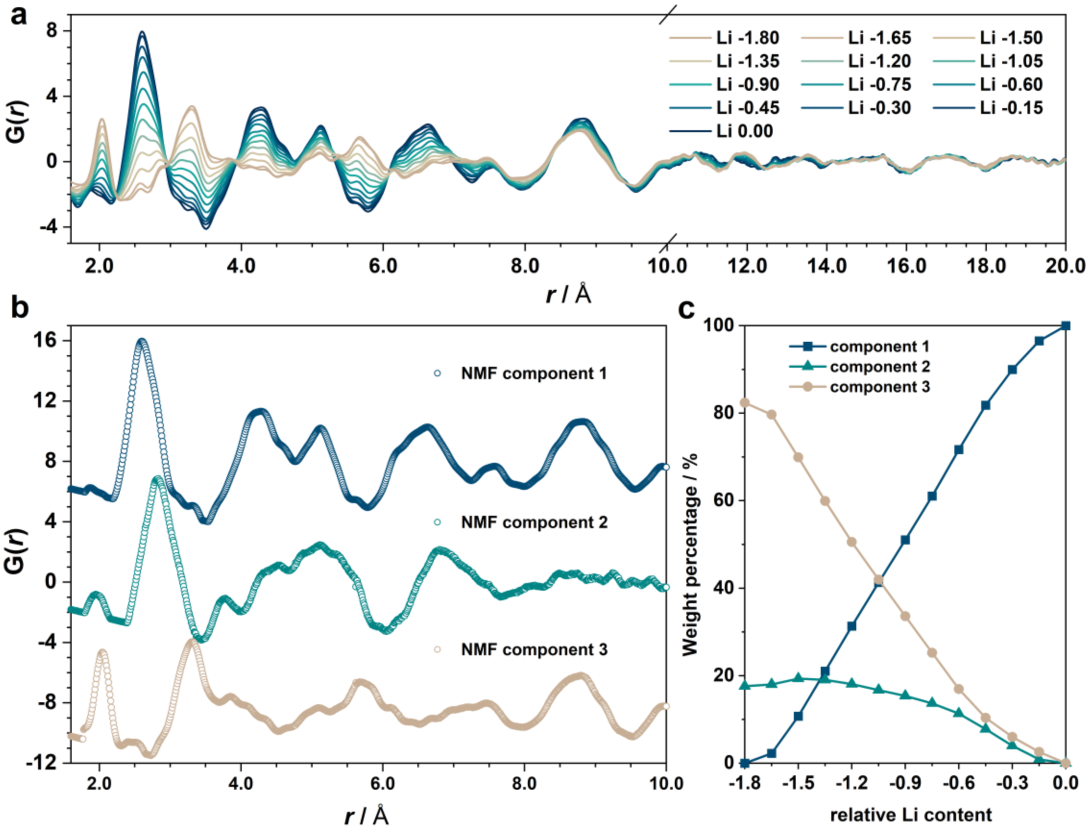

Supplementary Figure 16. a) *In situ* PDF patterns for Mn_3O_4 during the second discharge. Colour gradient from light brown ("Li -1.8") to dark blue ("Li 0.0") corresponds to an increasing concentration of Li. **b)** Three NMF-derived components show similar patterns comparing to their counterparts extracted from the first charge (Fig. 5b). **c)** Evolution of the NMF-derived weight percentage for the components shown in (b).

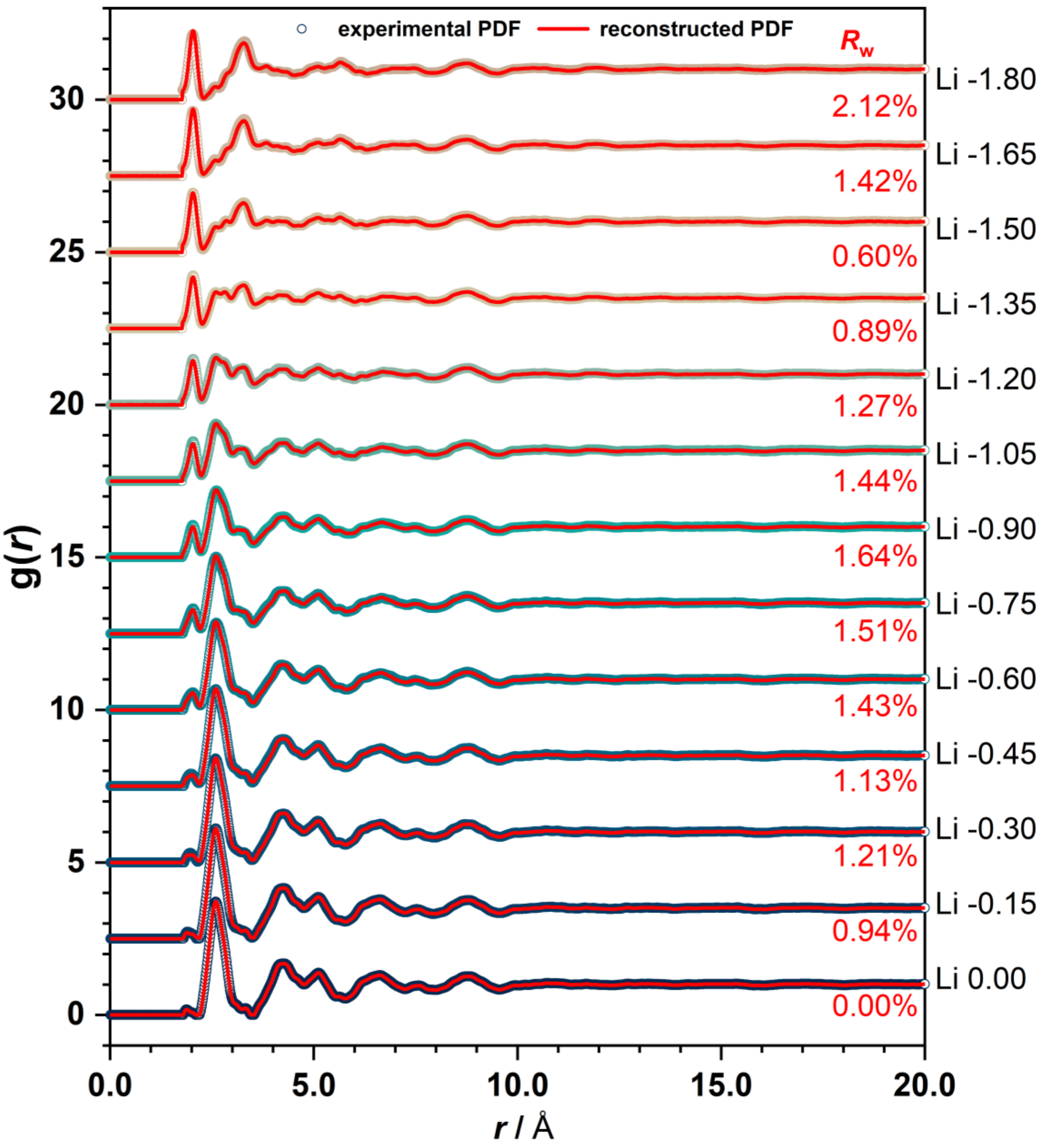

Supplementary Figure 17. Comparison between the experimental PDF from the second discharge (with the same colour gradient shown in Fig. S16) and the PDF reconstructed (red) using NMF-derived components and their respective weightings. R_w agreement factors are marked in red.

REFERENCES:

1 Xu, Y. *et al.* Superior electrochemical performance and structure evolution of mesoporous Fe_2O_3 anodes for lithium-ion batteries. *Nano Energy* **3**, 26-35 (2014).
