# Supplementary material for: Non-equilibrium metal oxides via reconversion chemistry in lithium-ion batteries
Source: Nat Commun. 2021 Jan 25;12:561. doi: 10.1038/s41467-020-20736-6 (PMC7835223; doi:10.1038/s41467-020-20736-6)
Supplement: Supplementary file 2 — Description of Additional Supplementary Files [file 41467_2020_20736_MOESM2_ESM.docx]

**Description of Additional Supplementary Files**

File Name: Supplementary Movie 1

Description: NMF analysis of the charging process of Mn_3_O_4_
